# The Association Between Emotional Intelligence and Academic Performance of Dental Students at King Saud University, Riyadh, Saudi Arabia

**DOI:** 10.7759/cureus.66431

**Published:** 2024-08-08

**Authors:** Mohammed I Alsaif, Abdulrahman Aljuni, Khalid Alyemni, Faisal Almuntashiri, Hebah M Hamdan, Hamdan Alamri, Rayan B Yaghmoor, Abdullah S Bin Rahmah

**Affiliations:** 1 Department of Periodontics and Community Dentistry, College of Dentistry, King Saud University, Riyadh, SAU; 2 Department of Dentistry, College of Dentistry, King Saud University, Riyadh, SAU; 3 Department of Preventive Dental Sciences, College of Dentistry, Majmaah University, Al Majma'ah, SAU; 4 Department of Restorative Dentistry, College of Dental Medicine, Umm Al-Qura University, Makkah, SAU

**Keywords:** saudi arabia, dental students, emotional intelligence, dental education, academic performance

## Abstract

Introduction: Emotional intelligence (EI) is reported to be associated with better academic performance. However, few studies from the Middle East have assessed whether EI affects academic performance in dental students. The objective of this study was to evaluate the relationship between EI and academic performance in a sample of Saudi Arabian dental students.

Methods: This cross-sectional, questionnaire-based study included first-year to fifth-year dental students and dental interns who were enrolled at King Saud University (KSU) in Riyadh, Saudi Arabia, for the 2023-2024 academic year. Eligible students were invited to complete the self-administered Schutte Self-Report Emotional Intelligence Test (SSEIT) and a demographic questionnaire between October 2023 and January 2024. Academic performance was assessed based on each student’s self-reported overall current grade point average (GPA) and was dichotomized into high GPA (between 4.5 and 5) and low GPA (less than 4.5).

Results: Of the 437 eligible students, 330 (75.5%) completed the questionnaires. The logistic regression analysis, after sequentially adjusting for various risk factors, showed significantly better academic performance for those who had higher EI (OR=2.6, P-value=0.02).

Conclusion. The findings of this study suggest a significant association between EI and academic success. EI is essential for improving academic performance in dental education.

## Introduction

Emotional intelligence (EI) can be described as an individual's capacity to understand and acknowledge both their own emotions and the emotions of others. This capacity includes adapting to difficult or stressful situations by managing and adjusting one's own feelings [1]. The field of dental education is widely recognized as one of the most rigorous and demanding areas of study, requiring dental students to develop a broad range of theoretical, clinical, and interpersonal competencies. This can be particularly challenging and stressful, as students must balance a demanding curriculum with the need to cultivate effective communication and teamwork skills [2].

It has been consistently reported that there is a considerable correlation between EI and various positive outcomes, including job satisfaction, interpersonal effectiveness, healthy relationships, job performance, psychological well-being, physical health, and psychophysiological measures of adaptive coping [3,4]. High EI scores have indicated a remarkable increase in capacity for understanding, regulating, and managing emotional stress. Therefore, individuals who score better on EI assessments are more likely to possess a strong sense of self-worth, achieve higher levels of professional success, and demonstrate effective leadership and management skills [2]. Academically, students who possess stronger EI skills are better equipped to succeed as they are more capable of regulating and controlling their emotions [5]. Research suggests that while EI and academic performance are linked, the relationship is indirect. Instead, several factors such as emotional well-being, motivation, and cognition mediate this connection. These intervening factors include metacognitive regulation as a learning strategy and a strong value placed on learning, in addition to overall emotional health [6].

Certain behaviors associated with exceptional professional practice in healthcare may be indicative of underlying abilities that are closely tied to EI [7]. EI is a critical factor in achieving effective practice, especially in the context of providing patient-centered care [8]. Studies have also shown that healthcare providers from different specialties who have higher EI tend to achieve better outcomes, both for themselves and their patients. It has been shown that EI among nurses buffers stress, reduces anxiety associated with end-of-life care, promotes effective communication, and improves performance [9].

Medical students who achieved excellent academic performance demonstrated higher EI scores compared to those with lower academic performance. Students who scored higher on EI assessments tended to have better exam grades and higher Grade Point Averages (GPAs) [10]. Another study from a medical college in Saudi Arabia concluded that EI was significantly linked with academic success [11]. In contrast, a study exploring the academic achievement of medical rehabilitation students showed no significant association between EI and GPAs [12]. Another study on dental hygiene students found that the EI subsets of self-control, motivation, and self-confidence were predictors of overall academic performance [13].

In dental education, studies have shown that students with higher EI tend to achieve better grades, demonstrate superior clinical skills, and exhibit more professional behavior [14-16]. Different studies reported that dental students with strong EI skills, such as stress management and effective communication, perform better academically [15,16]. In clinical settings, studies have shown that dental students with strong EI skills are associated with higher patient satisfaction and increased patient return rates [17,18].

Several studies have examined the factors influencing EI among dental students [3,4]. For example, a study in the Abha region of Saudi Arabia found that female dental students had slightly higher EI compared to male students. In the same study, EI was significantly higher among students who had better family support and those who reported enjoying studying dentistry [10]. Likewise, a study of Malaysian undergraduate dental students showed significantly higher EI skills among female students compared to male students and among students who selected dentistry out of personal interest rather than those who were influenced by others [19]. In a study that included only dental interns, it showed that factors like parents' education and physical exercise were significantly responsible for higher EI skills [20].

While there is growing evidence that EI benefits healthcare education and correlates positively with the skills of dental students, the link between EI and their academic performance remains unclear, with studies showing inconsistent findings. This study investigates the association between EI scores and academic performance among dental students at King Saud University, Riyadh, Saudi Arabia. Understanding this relationship is crucial, as it could offer valuable insights into how EI influences academic success. These insights could then inform strategies for enhancing dental education and student support programs. By clarifying the role of EI in academic performance, our findings may empower educators and administrators to develop targeted interventions that promote EI development. Ultimately, improving EI has the potential to benefit both educational outcomes and future professional success for dental students.

## Materials and methods

This was a questionnaire-based study conducted at the College of Dentistry, King Saud University, Riyadh, Saudi Arabia, from October 2023 to January 2024. The study was approved by the College of Dentistry Research Center (CDRC) at King Saud University (CDRC number: IRB 0483; reference number: KSU-HE-23-930). The study included all dental students from first year to fifth year and interns, both male and female, at King Saud University, Saudi Arabia. Participants who declined to participate were excluded.

Study tool and data collection

A self-administered questionnaire written in English was distributed among the participating students. The questionnaire contained 43 questions and was divided into two main sections: sociodemographic information and EI.

The sociodemographic section comprised nine questions regarding gender, academic year, parental education level, family support, smoking habits, physical activity level, sleep duration, interest in pursuing dentistry as a specialty, and GPA as a measure of academic performance.

EI was assessed through the Schutte Self-Report Emotional Intelligence Test (SSEIT) [21,22], a widely used and freely available measure, which had been validated previously. The SSEIT has 33 questions with total scores ranging from 33 to 165. Each question of the SSEIT is scored on a five-point Likert scale (1= strongly disagree, 2= disagree, 3= neither agree nor disagree, 4= agree, 5= strongly agree).

Prior to the main study, the questionnaire was pilot-tested on a sample of 10 participants. Dental students then self-administered the questionnaires at the dental college, following data collectors' notes and instructions provided at the beginning.

Statistical analysis

A significance level of p ≤ 0.05 was established to ensure the robustness of the results. The analysis employed descriptive statistics to summarize the data and logistic regression models to identify factors influencing the findings. Stata Statistical Software: Release 15/SE (2007; StataCorp LLC, College Station, Texas, United States) was used for analysis.

Numerical variables, including EI and GPA, were reported as mean ± standard deviation (SD). EI scores were distributed into five quintiles and GPA was dichotomized into high GPA (between 4.5 and 5) and low GPA (less than 4.5). The cutoff of 4.5 was used since it is the official university threshold for excellent and exceptional academic achievement.

EI scores were normally distributed and analyzed using independent samples t-tests for comparisons between two groups and one-way analysis of variance (ANOVA) for comparisons among three or more groups. On the other hand, due to the skewed nature of the GPA data, non-parametric tests including the Wilcoxon rank sum test for two groups and the Kruskal-Wallis test for three or more groups were used.

For logistic regression analysis, a progressive adjustment for potential confounders was performed, including gender, academic year, family support, parental education level, sleeping hours, physical activity, and smoking habits.

## Results

The study included 330 dental students from all academic years, including interns, at King Saud University, Riyadh, Saudi Arabia (Table 1). The response rate was 75.5%. Of the respondents, 201 (60.9%) were male and 129 (39.1%) were female. Interns comprised the largest group (27.8%), while first-year students had the lowest representation (10%). In terms of academic performance, 240 students (72.7%) had a high GPA (4.5 or higher out of 5), and 90 students (27.3%) had a low GPA (below 4.5). The overall mean GPA was 4.59 (SD = 0.31), with fifth-year students having the highest average (4.65) and fourth-year students the lowest (4.53). For the EI measure, scores showed no significant gender difference (average 125.5). However, there were variations across academic years. Year 3 students scored significantly (13.7 points) lower than Year 1 (p < 0.001), and Year 5 students scored significantly (10.9 points) higher than Year 3 (p < 0.001). Interestingly, while the high GPA group had a slightly higher average EI score (125.6) compared to the low GPA group (122.2), this difference was not statistically significant (p = 0.07).

**Table 1:** Descriptive statistics of the sample (N=330) for each variable by emotional intelligence. GPA: grade point average

Variable			GPA		Emotional Intelligence	
		n (%)	Mean (SD)	P-value	Mean (SD)	P-value
Gender	Male	201 (61)	4.55 (0.32)	0.32	125.5 (15.86)	0.25
	Female	129 (39)	4.65 (0.30)		125.5 (14.94)	
Year	1st	33 (10)	4.59 (0.30)	0.24	128.2 (11.59)	<0.001
	2nd	30 (9)	4.58 (0.24)		122.2 (19.12)	
	3rd	43 (13)	4.63 (0.27)		114.5 (15.43)	
	4th	48 (14.5)	4.53 (0.40)		122.4 (17.57)	
	5th	84 (25.5)	4.65 (0.25)		125.4 (11.36)	
	Intern	92 (28)	4.56 (0.35)		129.6 (14.71)	
Family support	Often	206 (62.5)	4.59 (0.32)	0.026	126.0 (14.82)	0.12
	Sometimes	95 (29)	4.63 (0.28)		123.0 (15.39)	
	Never	29 (0.5)	4.49 (0.34)		121.1 (18.47)	
Education level of the father	Master/PhD	94 (28)	4.62 (0.32)	0.53	125.2 (14.26)	0.86
	Bachelor’s	141 (43)	4.58 (0.28)		123.7 (15.21)	
	High school or less	95 (29)	4.60 (0.34)		124.7 (17.69)	
Education level of the mother	Master/PhD	67 (20)	4.56 (0.36)	0.28	124.0 (15.63)	0.63
	Bachelor’s	141 (43)	4.58 (0.29)		123.7 (15.77)	
	High school or less	122 (37)	4.62 (0.31)		126.3 (15.18)	
Sleeping Hours	Less than 6 hours	159 (48)	4.57 (0.31)	0.04	125.7 (17.07)	0.57
	More than 6 hours	161 (49)	4.62 (0.31)		123.7 (16.16)	
Physical activity	Very active	55 (16.5)	4.61 (0.3)	0.79	123.9 (19.12)	0.80
	Moderate	141 (42.5)	4.59 (0.32)		127.5 (14.19)	
	Not active	134 (40)	4.59 (0.31)		122.2 (14.44)	
Smoking	Yes	70 (21)	4.60 (0.31)	0.21	122.6 (18.44)	0.21
	No	260 (71)	4.56 (0.31)		125.3 (14.38)	
GPA	High (>= 4.5/5)	240 (72.7)	4.75 (0.13)	<0.001	125.6 (14.76)	0.07
	Low (< 4.5)	90 (27.2)	4.18 (0.28)		122.2 (16.48)	

**Figure 1:**
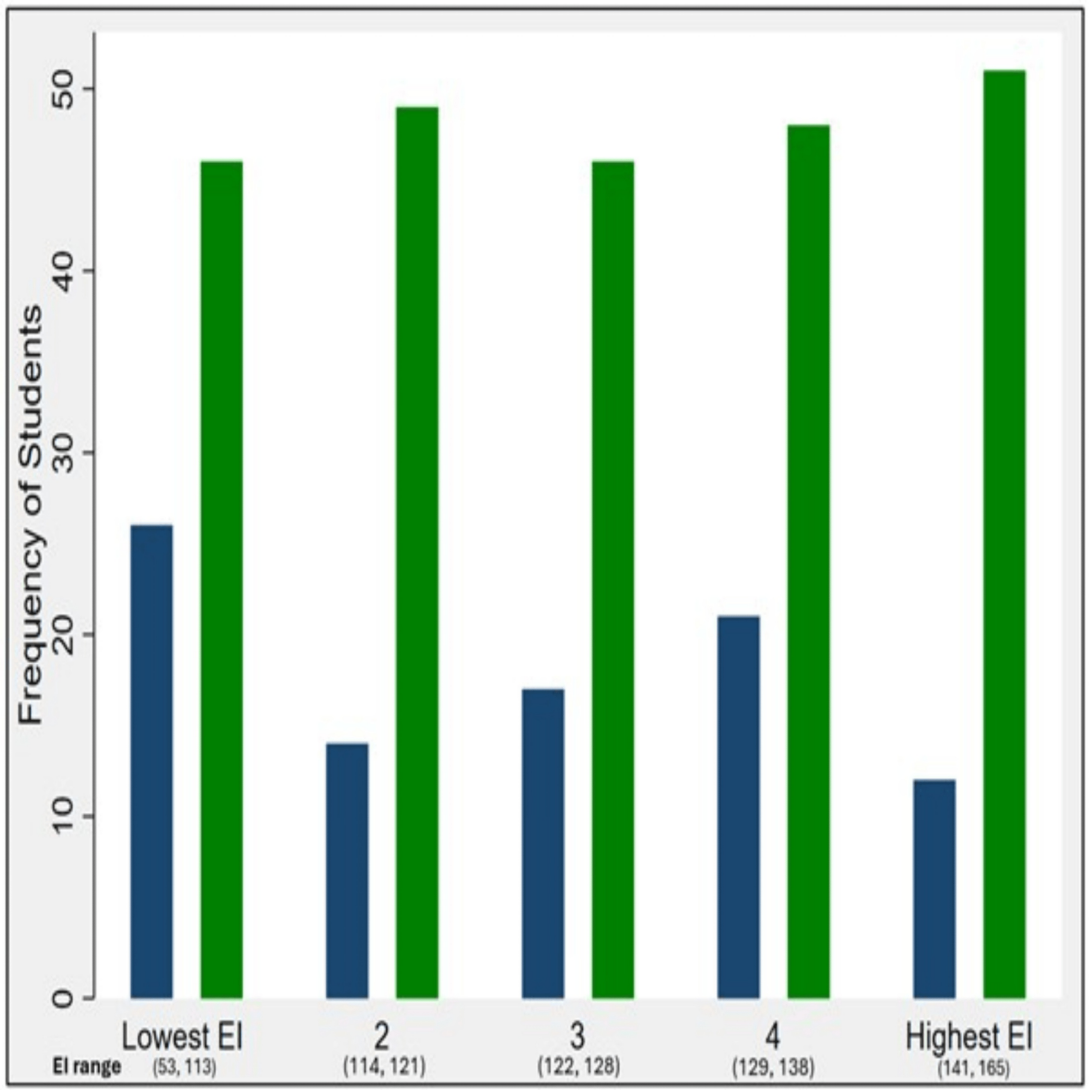
Frequencies of students (high versus low GPA) across quintiles of emotional intelligence. Navy blue: low GPA; Green: for high GPA. GPA: grade point average; EI: emotional intelligence

Analyzing the distribution of participants across the five EI score categories (quintiles) for the high and low GPA groups revealed an interesting trend (Figure 1). The high GPA group showed a gradual increase in the frequency of students as EI scores increased (inclining trend). Conversely, the low GPA group showed a decrease in frequency (declining trend). This suggests a potential association between higher academic performance and higher EI scores. However, a statistical test did not confirm this trend as significant (p-value = 0.12).

Our analysis using logistic regression (Table 2) revealed a positive association between EI and academic performance. Students in the highest EI category (quintile) were 2.4 times more likely to be in the high GPA group compared to those with lower EI scores (p-value = 0.03). Interestingly, when we adjusted for the academic year, the link between high EI and academic success became even stronger for the top EI group (odds ratio: 2.5, p-value = 0.02). However, the association weakened for students with lower EI scores.

Including gender in the analysis alongside academic year (model 3) did not alter the significant association for the top EI quintile. Students in this group maintained 2.5 times higher odds of being in the high GPA group compared to those with lower EI scores (OR = 2.5, P-value = 0.03). Furthermore, when family support and parental education were added to the model (model 4), the association for the top EI quintile actually strengthened further, remaining statistically significant (OR = 2.6, P-value = 0.02).

Even after accounting for additional factors like sleep quality, physical activity, and smoking habits (model 5), the analysis continued to reveal a significant association between higher EI and academic performance. Students in the high EI category remained 2.6 times more likely to be in the high GPA group compared to their counterparts with lower EI scores (OR = 2.6, p-value = 0.02).

**Table 2:** The multivariate logistic regression table of the association between EI quintiles and GPA groups (low versus high) (N=330). Model 1: Unadjusted; Model 2: Adjusted for year; Model 3: Model 2 + gender; Model 4: Model 3 + Family support and Parents education; Model 5: Model 4 + Sleeping, physical activity and smoking GPA: grade point average; EI: emotional intelligence

Emotional Intelligence	Model 1		Model 2		Model 3		Model 4		Model 5	
	OR (95%CI)	P-value	OR (95%CI)	P-value	OR (95%CI)	P-value	OR (95%CI)	P-value	OR (95%CI)	P-value
Lowest EI (Ref)										
2nd EI quintile	1.98 (0.92, 4.25)	0.08	1.89 (0.87, 4.08)	0.11	1.85 (0.84, 4.10)	0.13	1.97 (0.87, 4.42)	0.10	2.07 (0.90, 4.72)	0.09
3rd EI quintile	1.53 (0.73, 3.19)	0.26	1.48 (0.71, 3.11)	0.30	1.43 (0.66, 3.08)	0.37	1.28 (0.58, 2.83)	0.54	1.29 (0.57, 2.93)	0.54
4th EI quintile	1.29 (0.64, 2.61)	0.48	1.36 (0.67, 2.76)	0.40	1.27 (0.59, 2.72)	0.55	1.23 (0.56, 2.71)	0.60	1.26 (0.56, 2.84)	0.58
Highest EI	2.40 (1.09, 5.30)	0.03	2.52 (1.14, 5.61)	0.02	2.51 (1.09, 5.77)	0.03	2.67 (1.12, 6.36)	0.03	2.60 (1.07, 6.36)	0.04

## Discussion

This study builds upon existing research demonstrating that higher EI is associated with stronger social interactions and self-regulation skills, both of which are critical for academic success. Our findings echo this connection, revealing a significant positive association between EI and high GPA among dental students. This suggests that students with higher EI levels tend to achieve better academic results. These findings complement similar research conducted in nursing [9], medical [11], and dental education [9], all of which highlight a robust relationship between EI and academic performance.

Analyzing the findings regarding specific aspects of EI and their relationship with academic performance in multiple studies reveals both commonalities and divergences. For instance, higher EI consistently links to better stress management and communication skills, which contribute to a more positive learning environment and ultimately, academic success. However, the variability in how different EI factors influence academic performance in many contexts focuses on the complexity of this relation.

Our findings align with recent studies [10-12], all of which highlight a strong association between EI and academic performance. Similar to the previous study on medical students [10], our study observed that dental students with higher EI scores generally achieved higher GPAs, further reinforcing the link between EI and academic success.

Interestingly, our study, alongside other former studies [12,15], found similar average EI scores across academic years. This suggests that academic progression may not significantly influence EI levels in dental students. This mirrors findings in medical and nursing education, potentially indicating that students enter with high EI to cope with initial stress, and their EI development is then shaped by their experiences throughout their programs.

Higher EI scores observed among interns may suggest that the practical experience and maturity gained during their studies contribute to enhanced EI. Dental interns typically encounter a wider range of specialties with less direct academic supervision. These increased responsibilities and freedom likely prepare them for their future careers. This observed pattern aligns with other studies' proposition that real-life experience in clinical settings can significantly enhance EI [7,10].

Prior research suggests that females typically score higher on EI tests compared to males, often attributed to a greater tendency for emotional expression [15,18]. Notably, our study found no significant difference in EI scores between genders. This unexpected finding could be explained by two key factors. First, the demanding and homogeneous nature of dental education, with its rigorous curriculum and emphasis on specific skill sets, may lead to a more equal development of EI across genders. Second, the specific demands of dental education, with its unique challenges and stressors, might foster similar coping mechanisms and emotional skills among all students, regardless of gender. These factors suggest that within the structured and demanding environment of dental education, gender may play a less significant role in influencing EI. This aligns with contemporary research, which highlights a reduction in gender disparities in EI among students [12].

According to a previous study, there was a positive association between sleep duration and EI scores [16]. However, our findings did not reveal a significant relationship between sleep and EI. This discrepancy might be due to methodological differences between the studies or specific characteristics of our sample population.

Unlike a contemporary study that found a positive effect of parental education on student EI [20], our study did not observe a significant association between these two factors. This discrepancy might be due to variations in cultural contexts and sample characteristics. Cultural environments can shape how parental education impacts EI. For example, some cultures prioritize academic achievement, while others focus on developing emotional and social skills. Additionally, factors like socioeconomic status, the educational environment itself, peer influence, and personal experiences may have played a more prominent role in shaping the EI of this specific sample, potentially leading to less variability in EI scores.

Limitations

This study has some limitations that warrant consideration. The sample size was limited, and participants were recruited from a single university, potentially restricting the generalizability of the findings to other dental education programs. Additionally, the gender distribution was imbalanced, with a higher proportion of male participants (60.9%) compared to females (39.1%).

Furthermore, the study relied on GPA as a proxy for academic achievement, which might not fully capture the breadth of student accomplishments and skills. Another limitation is the cross-sectional design, which offers a single snapshot of the relationship between EI and academic performance. This design makes it difficult to establish causality, meaning we cannot definitively say that higher EI leads to better academic performance.

Finally, potential self-report bias in measuring EI and other variables could affect the accuracy of the data. Future research efforts could address these limitations by employing larger, more diverse samples, utilizing a longitudinal design to explore causal relationships, and incorporating more objective measures of both EI and academic achievement.

Implications for educational practice

By understanding the influence of EI on academic performance, we can develop targeted interventions to improve both student well-being and EI. Furthermore, the impact of specific variables can inform the creation of appropriate support plans for students struggling academically. These plans could incorporate various strategies, such as implementing EI training modules to equip students with skills to manage emotions, build relationships, and navigate social situations effectively. Additionally, fostering a learning environment that prioritizes both academic success and emotional well-being can create a supportive and positive classroom atmosphere. Finally, providing resources and activities that help students develop self-awareness, self-regulation, empathy, and other crucial emotional competencies can equip them with the tools they need to thrive. Various resources and activities have been proposed to enhance EI for students [23], including cognitive-behavioral therapy training [24], practical training [25], smartphone applications [26], and chatbot programs designed to simulate human conversation [27].

## Conclusions

The study highlights a robust correlation between EI and academic success among students in health-related fields. This emphasizes the necessity for further investigation into the components of EI, disciplinary variations, and mediating factors such as emotional well-being and learning strategies that could potentially enhance students' overall well-being and ultimately their academic achievement. Integrating EI training into the curricula of health specialties, particularly dentistry, is anticipated to not only improve academic performance but also positively impact future clinical practice. Such integration could equip future healthcare professionals with the necessary emotional and interpersonal skills to excel in patient-centered care environments.
